# Markers of T cell activation and exhaustion in plasma are associated with persistent symptoms up to 18 months following mild SARS-CoV-2 infection

**DOI:** 10.3389/fimmu.2025.1578208

**Published:** 2025-05-30

**Authors:** Thor Ueland, Rebecca Jane Cox, Annika E. Michelsen, Elisabeth Berg Fjelltveit, Kari Otterdal, Tuva Dahl, Fan Zhou, Rebecca Elyanow, Pål Aukrust, Bjørn Blomberg, Bente E. Halvorsen, Nina Langeland

**Affiliations:** ^1^ Research Institute for Internal Medicine, Oslo University Hospital Rikshospitalet, Oslo, Norway; ^2^ Institute of Clinical Medicine, University of Oslo, Oslo, Norway; ^3^ Thrombosis Research Center (TREC), Division of Internal Medicine, University Hospital of North Norway, Tromsø, Norway; ^4^ Department of Microbiology, Haukeland University Hospital, Bergen, Norway; ^5^ Influenza Centre, Department of Clinical Science, University of Bergen, Bergen, Norway; ^6^ Department of Clinical Science, University of Bergen, Bergen, Norway; ^7^ Adaptive Biotechnologies, Seattle, WA, United States; ^8^ Section of Clinical Immunology and Infectious Diseases, Oslo University Hospital Rikshospitalet, Oslo, Norway; ^9^ National Advisory Unit for Tropical Infectious Diseases, Haukeland University Hospital, Bergen, Norway

**Keywords:** post-covid condition, T cell activation and exhaustion, persistent symptoms, long-term follow-up, SARS-CoV-2, mild covid-19 infection

## Abstract

**Background:**

Persistent symptoms following SARS-CoV-2 is an increasing problem after COVID-19 disease. The pathogenesis of this persistent post Covid-19 Condition (PCC) is, however, largely unknown. We hypothesized that persistent T cell activation and exhaustion play a role in PCC development.

**Methods:**

We examined plasma levels of soluble (s) CD25, TIM-3 and LAG-3, all markers of T cell activation/exhaustion, by enzyme immunoassays in 170 home-isolated and 53 hospitalized patients for up to 18 months after COVID-19 in relation to persistent symptomatology.

**Results:**

Our major findings were: (i) Cases with persistent dyspnea and fatigue had markedly higher sCD25 at 6–18 months with a more modest increase in sTIM-3. (ii) Cases with memory problems at 12–18 months had increased sLAG-3 iii) sCD25 correlated with SARS-CoV-2 antibody titers and microneutralization titers only in cases with PCC while sTIM-3 correlated with these parameters irrespectively of symptoms. iv) Although hospitalized patients had markedly elevated levels of T cell activation/exhausting markers during follow-up, there was no relation to PCC symptoms.

**Conclusion:**

Our study indicates a role for T cell activation/exhaustion in PCC following home isolated COVID-19 infection, with somewhat different patterns of sCD25, sTIM-3 and sLAG-3, but not in hospitalized COVID-19 patients where disease severity may be more important.

## 1 Introduction

According to the World Health Organization (WHO) there have been over 775 million SARS-CoV-2 cases and 7.0 million deaths due to COVID-19 recorded since December 2019 (1). Notably, over 40% of cases develop symptoms diagnosed as post-COVID Condition (PCC) in systematic reviews (2, 3). There is growing awareness that the incidence of PCC will have a significant impact on the global society for many years to come, including immense health care costs. However, the pathogenesis of PCC is incompletely understood, which hampers the development of treatment and prevention modalities that are so far almost non-existent (4).

Both innate and adaptive immune responses play an essential role in protection from SARS-CoV-2 infection but may be detrimental to the host when dysregulated. Indeed, overactive and persistent immune responses due to viral or host factors seem to be involved in the development and severity of long-term sequelae, like PCC, following SARS-CoV-2 infection (4–6). However, the involvement of immune activation in the development of PCC is far from clear as most studies have short-term follow-up, lack longitudinal testing and are often dominated by hospitalized patients.

T cell activation has been implicated in the pathogenesis of PCC including a role for specific T cell subsets and T cell receptor profiles, as well as T-cell derived cytokines like interleukin (IL)-4, IL-7 and IL-17 (7–9). We have previously shown persistently elevated circulating levels of T-cell immunoglobulin and mucin domain-3 (TIM-3), a potential marker of T cell exhaustion, for up to 12 months after hospitalization for acute COVID-19 disease. Moreover, TIM-3 levels at three months after hospitalization were associated with pulmonary pathology at this time (10). However, data on home-isolated patients and longitudinal data on both T cell markers and symptomatology on PCC are scarcer.

We have previously reported the presence of PCC in a well-characterized population of home-isolated COVID-19 cases, representing approximately 90% of all patients confirmed infected with SARS-CoV-2 in the municipality of Bergen, Norway between 28 February and 4 April 2020 (11, 12). The patients were followed with longitudinal blood sampling and symptom scores of PCC for up to 18 months. We hypothesized that persistent T cell activation could be related to PCC and this hypothesis was tested in our cohort by analyzing plasma levels of soluble (s) sCD25, sTIM-3 and lymphocyte-activation gene 3 (LAG-3), reflecting various aspects of T cell activation/exhaustion, and their relation to PCC symptomatology during follow-up. For comparison we also included 53 hospitalized COVID-19 patients where blood samples were collected for up to18 months after hospitalization when symptom scores of PCC also were registered.

## 2 Materials and methods

### 2.1 Study population

The study population has previously been described (11, 12). Cases included home-isolated patients with reverse transcription-polymerase chain reaction (RT-PCR) confirmed SARS-CoV-2 infection, tested at the city’s centralized testing facility (Bergen Municipality Emergency Clinic) between 28 February 2020 and 4 April 2020 (13), and those admitted to the two neighboring city hospitals: Haukeland University Hospital and Haraldsplass Deaconess Hospital. All home isolated patients had mild disease, and none were hospitalized at any time-point during follow-up. All participants were infected with the Wuhan variant. The length of the hospitalization for the hospitalized patients differed, but all were discharged within the recruitment period (i.e., before 4. April 2020). The hospitalized patients were categorized according to severity, with the severity scoring system used by Beigel JH et al. (13). None of the hospitalized patients were asymptomatic or without medical needs, 48% had medical needs without oxygen, and 11% had oxygen need at some level including respirator support. In the present sub-study, we excluded household members of the primary home-isolated cases to avoid inclusion of children and to make the cohort more homogenous. Thus, of the original study population, we included 170 home-isolated participants who all were sampled at 2, 6 and 12 months and 147 had an additional sample at 18-month follow-up. This study population of home-isolated participants and 53 hospitalized patients with available blood samples is described in **Table 1**. For hospitalized patients, we accepted longer inclusion due to delay in hospitalization, up to 6 May. Blood samples and questionnaires were collected at several time-points up-to 18 months of follow-up.

**Table 1:** Demographic and clinical profile of COVID-19 cases.

demographic	Non-hospitalized COVID-19 positive (n=170)	Hospitalized COVID-19 positive (n=53)	*p*-value
Age	45 ± 16	56 ± 15	<0.001
Male sex	90 (53%)	23 (43%)	0.23
BMI	25.0 ± 3.4	28.0 ± 5.4	<0.001
Comorbidities*	71 (42%)	38 (72%)	<0.001
Total symptom prevalence at month 6/12/18	74 (44%)/80 (47%)/53 (45%)	40 (76%) at 6mo.	<0.001**
Available blood samples at month: 2/6/12/18	170/170/169/127	53/44/53/46	0.57

BMI, body mass index. *Comorbidities include asthma, chronic obstructive pulmonary disease, hypertension, chronic heart disease, rheumatic disease, diabetes, cancer, neurological disease, immunosuppressive conditions, or other severe or chronic disorders. ** comparing symptom prevalence at 6 months.

### 2.2 Ethical considerations

The study was approved by the Regional Ethics Committee of Western Norway (#118664). All eligible individuals received both oral and written information about the study protocol and provided written informed consent on inclusion.

### 2.3 Clinical data collection

Participant data was entered into electronic case report form (using the Research Electronic Data Capture database REDCap, Vanderbilt University, Nashville, TN) software and subsequently stored on a secure research server. All cases recruited at Bergen Municipality Emergency Clinic were followed up for 18 months with systematic interviews at 6, 12 and 18 months, and blood samples were collected at 2, 6, and 12 months. A total of 116 cases had an additional follow-up with blood samples and systematic interviews at 18 months. The hospitalized patients were followed for a similar duration with blood sampling at each time-point, however, systematic interviews were only assessed at 6 months for this group. Comorbidities recorded and used for adjustment were the composite of asthma, chronic obstructive pulmonary disease, hypertension, chronic heart disease, rheumatic disease, diabetes, cancer, neurological disease, immunosuppressive conditions, or other severe or chronic disorders as previously reported (11). Symptoms related to fatigue and cognitive symptoms were recorded by the validated Chalder Fatigue Scale (CFS) (14) which is also validated in the Norwegian general population (15). PCC was defined as persistent or new onset symptoms 3 months after SARS-CoV-2 infection (11, 12). All home-isolated and hospitalized patients with available serum samples were included, regardless of if they had PCC. In the present study symptoms were assessed at 6-, 12-, and 18-months, using a dichotomized yes/no questionnaire for persisting dyspnea, sleep problems, headache, dizziness, tingling, palpitations, gastrointestinal problems, or low-grade fever. The validated Chalder Fatigue Scale (CFS) questions 1, 8, and 11 were used for symptoms of fatigue, impaired concentration, and memory problems using a bimodal score. The prevalence of fatigue, impaired concentration, and memory problems was derived from the corresponding bimodal score of the CFS item 1, 8, and 11, respectively. The severity of dyspnea was also recorded as a bimodal score.

### 2.4 Blood sampling protocol and measurements of soluble markers of T cell activation/exhaustion

Serum samples were separated and aliquoted before storage at -80°C. Serum levels of sCD25, sTIM-3 and sLAG-3 were measured in duplicate by enzyme-linked immunosorbent assay (ELISA) using commercially available antibodies (R&D Systems, Minneapolis, MN) in a 384-format using a combination of a SELMA pipetting robot (Analytik Jena AG, Jena, Germany) and a BioTek dispenser/washer (BioTek Instruments, Winooski, VT). Absorption was read at 450 nm by using an enzyme immunoassay plate reader (BioTek Instruments) with wavelength correction set to 540 nm. Samples from home isolated and hospitalized patients were run on the same 384-well plate; the intraassay coefficients of variation for sCD25, sTIM-3, and sLAG-3 were 5.4%, 3.6%, and 8.0%, respectively.

### 2.5 Analyses of IgG against SARS-CoV-2 spike protein

Serum samples were analyzed in a two-step ELISA, firstly by antibody screening for the Wuhan receptor-binding domain (RBD), followed by Wuhan spike ELISA using the Wuhan spike protein, 100ng/well as previously described (16, 17).

### 2.6 The microneutralization assay

The microneutralization assay was performed in a certified Biosafety Level-3 Laboratory using a local SARS-CoV-2 isolate from March 2020, hCoV-19/Norway/Bergen-01/2020 (GISAID accession ID EPI_ISL_541970) as previously described (16, 17).

### 2.7 Identification of SARS-CoV-2-associated T-cell receptor β sequences

Genomic DNA was extracted from EDTA blood using the Qiagen DNeasy Blood Extraction Kit (QIAGEN, Germantown, MD) and amplified in a bias-controlled multiplex PCR, followed by high-throughput sequencing. SARS-CoV-2-associated CDR3 regions of TCRβ chains were sequenced using the ImmunoSEQ Assay T-MAP COVID platform (Adaptive Biotechnologies, Seattle, WA) as previously described (18). The clonal breadth was defined as the relative number of SARS-CoV-2-associated TCR clonal lineages in a repertoire, and the relative expansion of SARS-CoV-2-associated TCR clonotypes was defined as the clonal depth.

### 2.8 Statistical analyses

Plasma levels of T cell activation/exhaustion markers (log10 transformed) were compared at each time-point between symptom groups or between hospitalized and home-isolated cases using multivariate GLM with T-cell markers as dependent, case or symptom as fixed factor and age, sex and comorbidity as covariates. The temporal trajectory of the T cell markers according to severity (i.e., hospitalized vs. home isolated) was analyzed by linear mixed models using untransformed complement levels and a log link function. The subject was used as random effect and severity and time as fixed effects (also as interaction) and age, sex and comorbidities as covariates. Spearman’s Rank-Order Correlation was used to assess correlations. P-values are two-sided and considered significant when <0.05.

## 3 Results

### 3.1 Demographic and clinical characteristics of the study cohort

Demographic data and clinical characteristics from the two groups are given in **Table 1**. Compared to hospitalized cases (n=53), the home-isolated cases (n=170) were younger (mean ± standard deviation: 45 ± 16 years vs. 56 ± 15, p<0.001), had lower body mass index (25.0 ± 3.4 vs. 28.0 ± 5.4, p<0.001) and fewer comorbidities (42% vs. 72%, p<0.001) compared to hospitalized patients. Further, hospitalized patients had markedly more symptoms with 76% reporting one or more symptoms at 6-month follow-up compared to 44% in home-isolated patients (p<0.001). All hospitalized patients were discharged within the recruitment period.

### 3.2 T cell activation/exhaustion markers in relation to persisting symptoms in home-isolated COVID-19 cases

**Figure 1:**
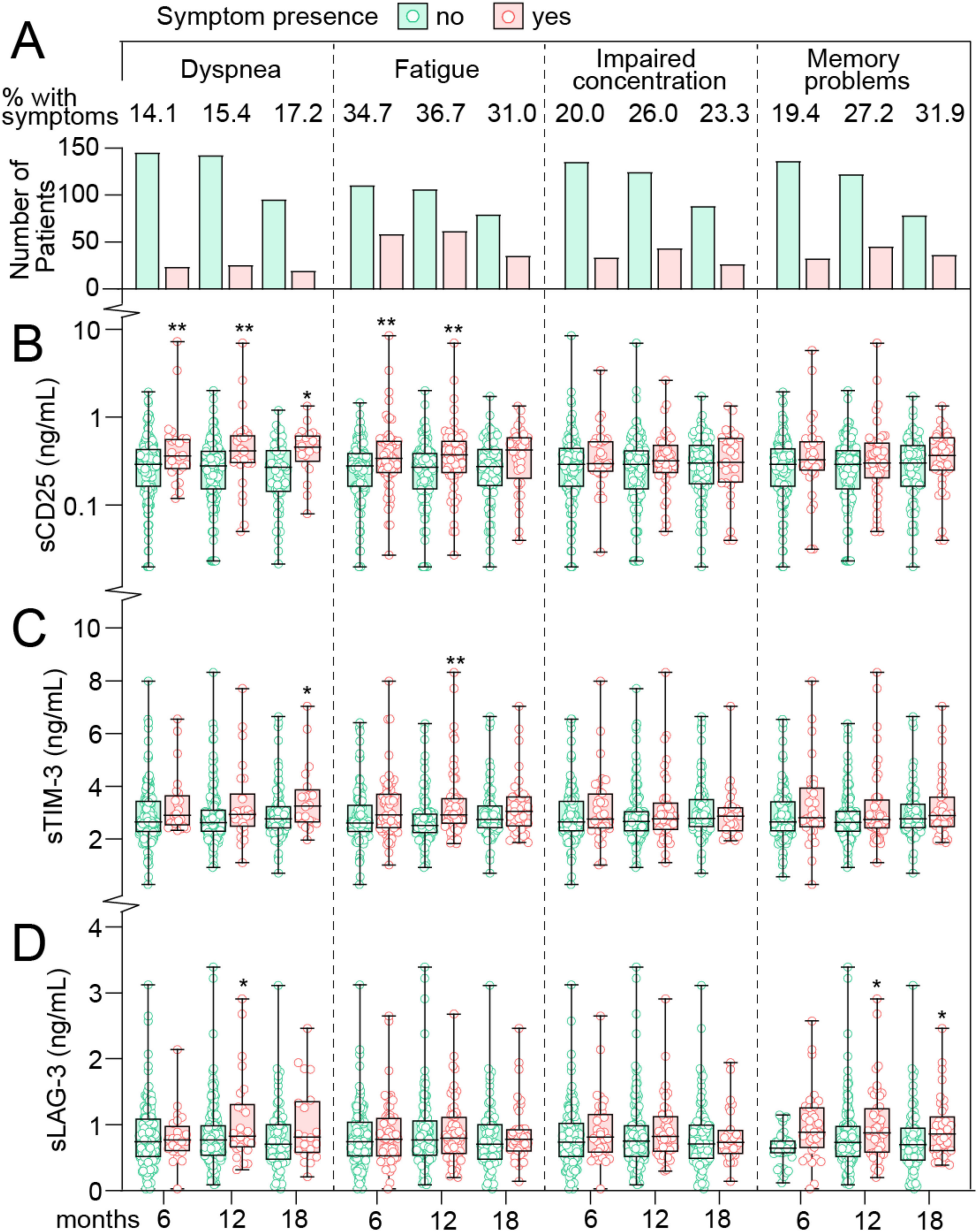
T cell activation markers in home-isolated COVID-19 cases during long-term follow-up in relation to persisting symptoms. **(A)** prevalence of symptoms at 6, 12 and 18 (x-axis) months after infection. Numbers at the top reflect the percentage of patients with symptoms at each time-point. Tukey plots showing plasma levels of **(B)** soluble (s)CD25 **(C)** T-cell immunoglobulin and mucin domain 3 (sTIM-3) and **(D)** Lymphocyte-activation gene 3 (sLAG-3) in relation to symptoms at 6, 12 and 18 months after infection. *p<0.05, **p<0.01 *vs.* no symptoms. Data were analyzed by multivariate GLM adjusted for age, sex and comorbidities.

We first focused on T cell markers in relation to PCC symptoms in mild COVID-19 cases where T cell markers and symptom scores were analyzed at the same time-point. As shown in **Table 1**, symptom prevalence was stable across the three time-points from 6 to 18 months with 44-47% reporting at least one or more symptoms. Likewise, the prevalence of specific symptoms in these home-isolated patients was relatively stable across these time-points as shown in **Figure 1A**. The prevalence of dyspnea was 14-17%, fatigue 31-37%, impaired concentration was registered in 20-26% of cases, and memory problems in 19-32%. **Figures 1B–D** shows the T cell markers in relation to having, or not having persistent symptoms on the selected scales with covariate adjusted (i.e., age, sex, comorbidities) with estimated marginal means (EMMs) shown in **Supplementary Table 1**. sCD25 was significantly higher in patients who experienced dyspnea with EMMs at all-time points (6, 12 and 18 months), i.e., 60-74% higher in cases who experienced dyspnea. A similar trend was observed for fatigue, where sCD25 was significantly higher (52%) at both 6 and 12 months compared to cases without symptoms. No association between sCD25 and neurocognitive problems was observed.

sTIM-3 levels were modestly higher (~10%) in cases who experienced dyspnea and fatigue at most time-points, reaching statistical significance at 18 months in relation to dyspnea (15% higher), and at 12 months in cases with fatigue (16% higher). No association between sTIM-3 and neurocognitive problems was observed.

sLAG-3 levels were not different between cases with and without physical symptoms or impaired concentration, but notably, sLAG-3 were significantly higher at 12- (26%) and 18-months (44%) in cases who experienced memory problems.

### 3.3 T cell activation/exhaustion markers in home-isolated COVID-19 individuals in relation to composite scores of persisting symptoms

**Figure 2:**
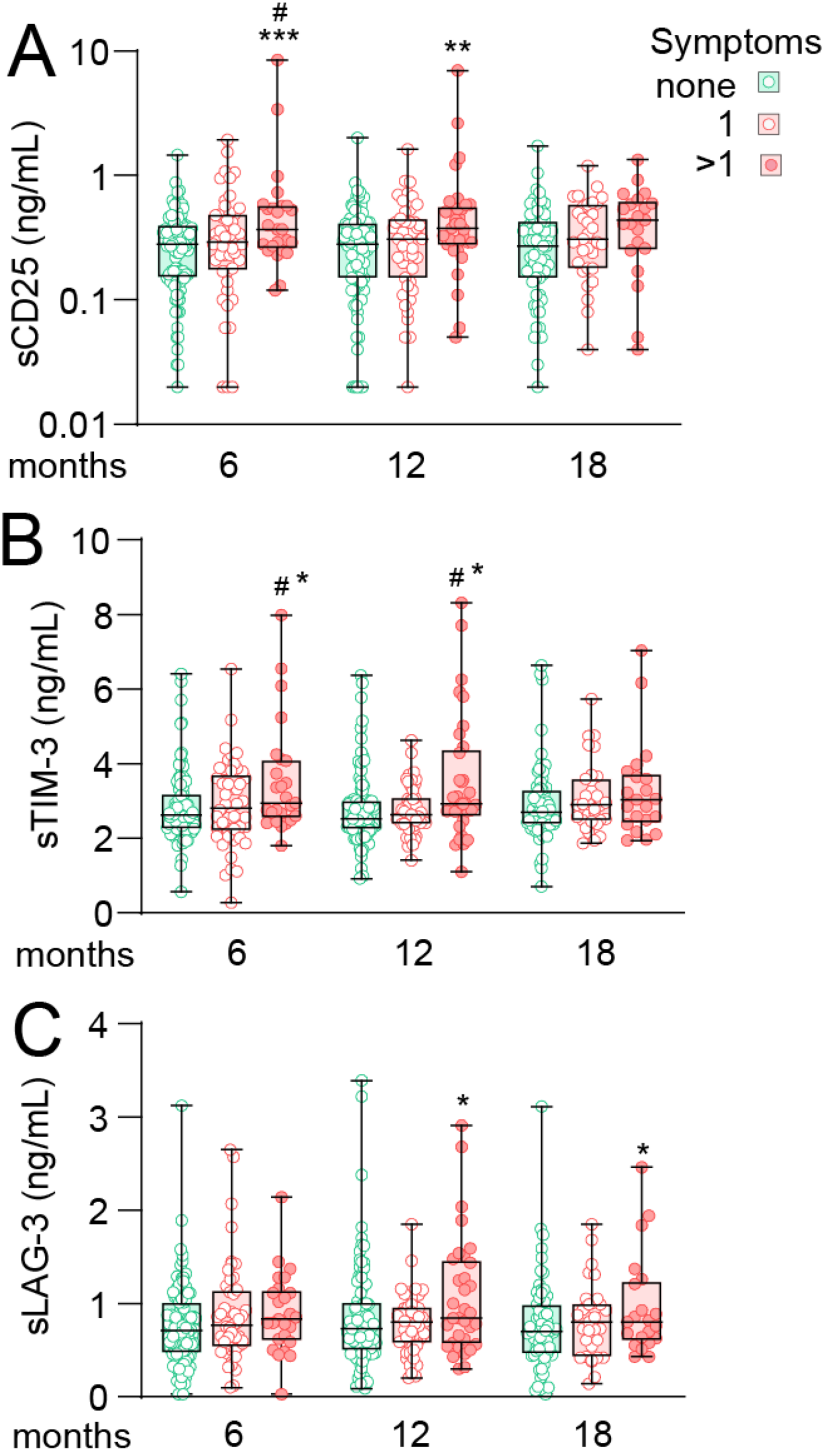
T cell activation markers during long-term follow-up in home-isolated COVID-19 cases in relation to composite scores of persisting symptoms. Tukey plots showing plasma levels **(A)** soluble (s)CD25 **(B)** T-cell immunoglobulin and mucin domain 3 (sTIM-3) and **(C)** Lymphocyte-activation gene 3 (sLAG-3) in relation to composite scores of symptoms at 6, 12 and 18 months after infection. Cases were grouped as having no symptoms (green), one symptom (of dyspnea, fatigue, memory problems or impaired concentration, lighter red) or two or more symptoms (darker red). *p<0.05, **p<0.01 vs. no symptoms; #p<0.05 vs. one symptom. Number of observation for 0/1/>1 symptoms was 96/49/25 at 6 months, 90/47/33 at 12 months and 64/31/22 at 18 months. Data were analyzed by multivariate GLM adjusted for age, sex and comorbidities. ***p<0.001.

We next examined if the level of T cell markers were associated with symptom burden as reflected by composite scores categorizing symptoms as having no symptoms, 1 symptom or 2 or more symptoms of the selected symptom scales (i.e., dyspnea, fatigue, impaired concentration, memory problems). Data for sCD25 are shown in **Figure 2A**, with adjusted EMMs presented in **Supplementary Table 2**, demonstrating that cases with ≥2 symptoms at 6 months had EMMs that were 88% (p<0.001) and 60% (p<0.05) higher than cases without- or with 1-symptom, respectively. At 12 months follow-up, cases with >1 symptoms had 75% higher levels of sCD25 (p<0.01) with a similar trend for this group at 18 months (50% higher, p=0.055).

Although with less perceptual difference between groups, a similar trend as for sCD25 was observed for sTIM-3 at 6- and 12-month follow-up (**Figure 2B**), with higher levels in cases with ≥2 symptoms compared to both no symptoms (18%, p<0.05) and cases with 1 symptom (21%, p<0.05) at 6 months with a similar profile at 12 months.

sLAG-3 levels were similar between groups at 6 months but interestingly, at 12 and 18 months, EMMs were 32% and 43% higher (both p<0.05), respectively, in cases with >1 symptoms compared to those without symptoms.

### 3.4 Associations between T cell activation/exhaustion markers, antibody titers and T cell clonal response

**Figure 3:**
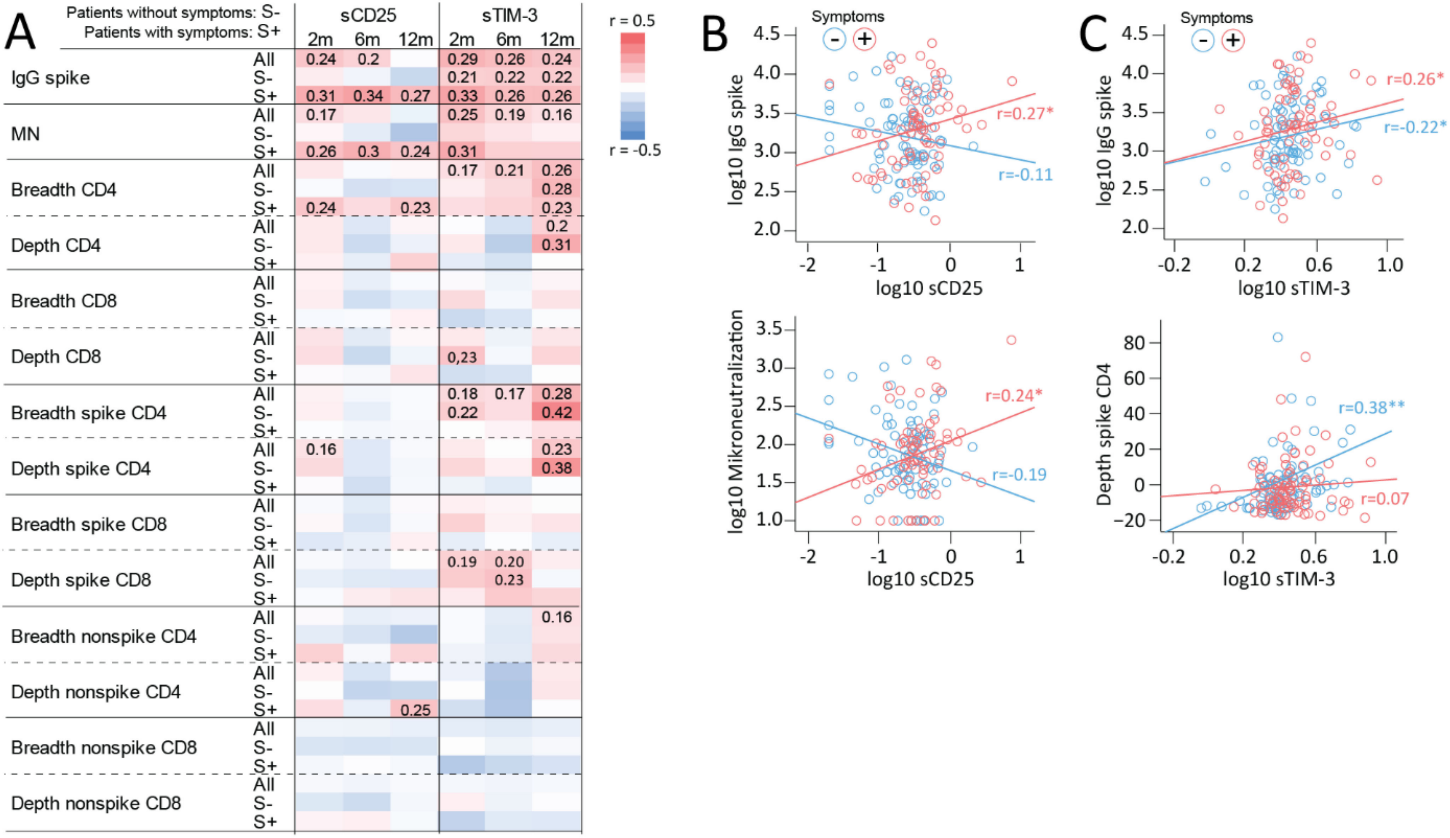
Associations between T cell activation/exhaustion markers, antibody titers and T cell clonal response. **(A)** Heatmap showing correlations between sCD25 and sTIM-3 and IgG spike and microneutralization titers and TCRβ SARS-CoV-2 spike specific and non-spike specific CD4^+^ and CD8^+^ T cell clonal depth and breadth at different time-points during follow-up in all home-isolated COVID-19 cases (All: in all patients) and in relation to having (S+) or not having any symptom (S-) at the same time-points. For correlations at 2 months, 6-month symptoms were used. Spearman correlation coefficients are included for significant correlations. Selected correlations plots at 12 months reflecting main findings for **(B)** sCD25 showing positive correlation with IgG spike and microneutralization titers in cases with symptoms and **(C)** sTIM-3 showing positive correlations with IgG spike titers irrespective of symptom burden and SARS-CoV-2 spike specific CD4^+^ clonal breadth in participants without symptoms. *p<0.05, **p<0.01.

SARS-COV-2 spike-specific IgG antibody titers and T cell clonal responses, i.e., the spike-specific clonal CD4^+^ T-cell receptor depth, have previously been related to persisting symptoms in these cases (11) and are shown at different time-points and in relation symptoms in **Supplementary Table S3**. We next assessed the association between these indices and circulating levels of T cell markers in all home-isolated cases as well as separately in cases with and without PCC symptoms. As shown in the heatmap in **Figure 3A**, sCD25 correlated positively with SARS-COV-2 spike-specific IgG antibody titers at 6–8 weeks and 6 months, as well as with microneutralization titers at 2 months when all cases were considered. However, these correlations were not present in patients without symptoms but restricted to the group experiencing at least 1 symptom at 6–8 weeks, 6 and 12 months. Correlation plots at 12 months are shown in **Figure 3B**. In relation to TCR, sCD25 correlated poorly with SARS-CoV-2 spike specific and non-specific clonal depth and breadth for CD4^+^ and CD8^+^ T cells although a correlation with the depth of CD4^+^ T cell non-spike specific responses in participants with symptoms was noted.

sTIM-3 correlated positively to SARS-CoV-2 spike-specific IgG antibody titers at 6–8 weeks, 6 and 12 months, but in contrast to sCD25, with similar coefficients in cases with and without symptoms, as shown for 12-month data in **Figure 3C**. In line with this, a positive correlation with microneutralization titers was observed in all cases (i.e., irrespective of symptoms). Regarding correlations between sTIM-3 and T cell clonal responses, some patterns stood out. First, sTIM-3 correlated positively with CD4^+^ T cell clonal breadth responses at 6–8 weeks, 6 and 12 months, with stronger correlations at 12 months. Second, a similar pattern, was observed for SARS-COV-2 spike-specific CD4^+^ T cell clonal breadth and depth in all cases, however, largely driven by non-symptomatic participants as shown in **Figure 3C** (bottom panel). Third, like the pattern for sCD25, there were in general few correlations of sTIM-3 with clonal CD8^+^ T cell response except for a correlation with SARS-CoV-2 spike specific CD8^+^ T cell clonal depth at 6–8 weeks and 6 months in all cases and participants without symptoms. No correlations were observed for sLAG-3 (**Supplementary Figure 1**).

### 3.5 T cell activation markers during long-term follow-up in hospitalized COVID-19 patients and relation to persisting symptoms at 6 months

**Figure 4:**
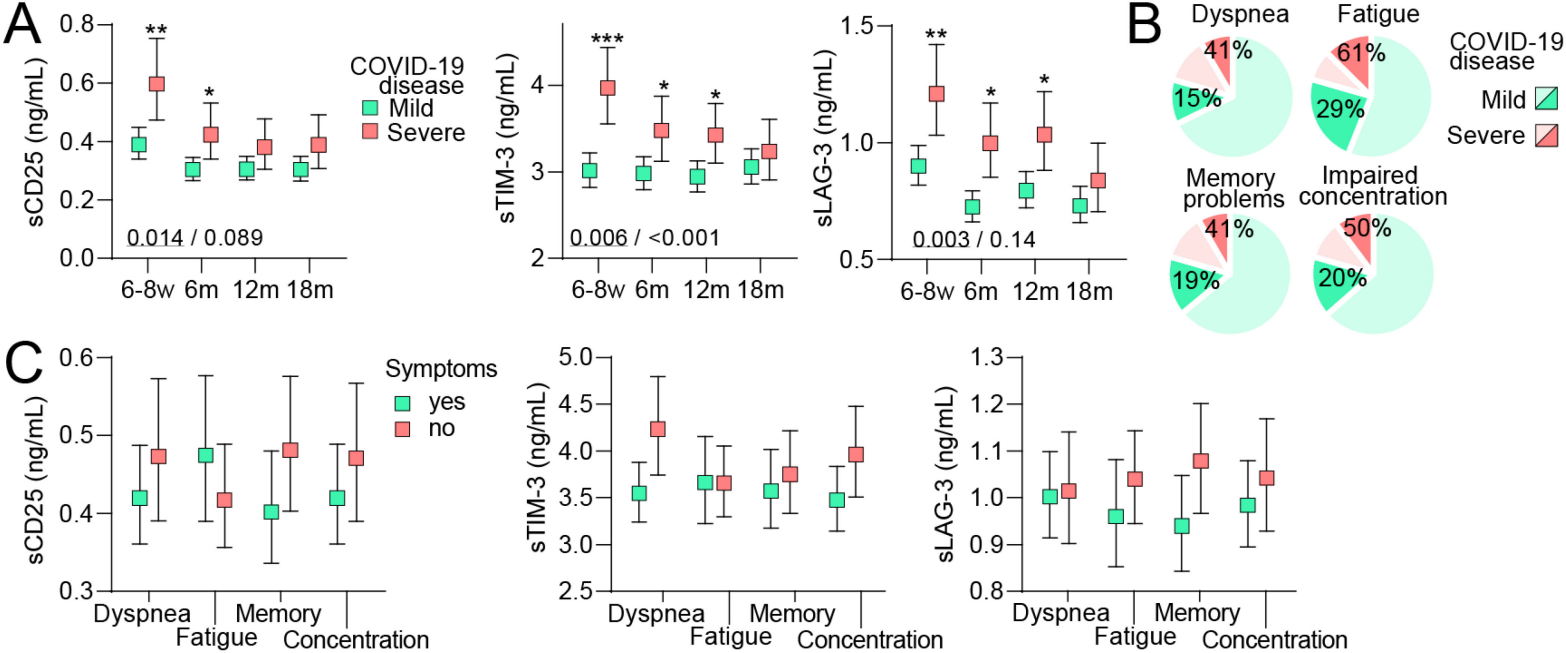
T cell activation markers during long-term follow-up in relation to persisting symptoms in mild and severe COVID-19 disease. **(A)** Plasma levels of soluble (s)CD25, T-cell immunoglobulin and mucin domain 3 (sTim-3) and Lymphocyte-activation gene 3 (sLAG-3) during 18 months of follow-up following mild (home-isolated COVID-19 cases) or severe (hospitalized patients) COVID-19 disease. Data are shown as estimated marginal means with 95% CI. Written p-values reflect the group (underscored) and group*time effect from the linear mixed model analysis. *p<0.05, **p<0.01, p<0.001, ***p<0.001 vs. mild disease. **(B)** prevalence of symptoms 6 months after infection in individuals who had mild (darker green) or severe (darker red) COVID-19 disease. **(C)** Plasma levels of T cell activation markers at 6 months of follow-up following severe (hospitalized patients) COVID-19 disease in relation to having symptoms or not. Data were analyzed by multivariate GLM. All analysis were adjusted for age, sex and comorbidities.

Finally, we compared T cell activation/fatigue markers in home-isolated and hospitalized COVID-19 cases (**Figure 4A**). Within the hospitalized patients, only 11% had severe (need for oxygen supplementation) compared to moderate disease severity and in our opinion, there were too few individuals to make comparisons within this patient group. For all markers, significantly higher initial levels were observed in severe cases followed by a decline, but levels remained higher than in home-isolated patients up to 12 months for sTIM-3 and sLAG-3 and 6 months for sCD25. The higher total symptom prevalence in severe cases at 6 months was seen for all symptom scales (**Figure 4B**). However, we found no significant differences in levels of either sCD25, sTIM-3 or sLAG-3 between hospitalized COVID-19 cases with and without symptoms across any symptom scale (**Figure 4C**). Of note, due to the limited number of severe cases and potentially more augmented regulation, the confidence intervals for the estimated marginal means in the symptom-scale subgroup analysis were large.

## 4 Discussion

We have recently demonstrated a high burden of persisting symptoms that correlate with SARS-CoV-2-specific immune responses in this population of home-isolated COVID-19 cases during long-term follow-up. The present study extends these findings by showing that circulating markers of T cell activation/exhaustion were significantly associated with persistent PCC, also after adjusting for relevant confounders. We found that: i) cases with persistent dyspnea and fatigue had markedly higher sCD25 at 6–18 months with a more modest increase in sTIM-3; ii) cases with memory problems at 12–18 months had increased sLAG3; iii) patients with a high symptom burden (i.e., >1 symptom) had higher sTIM-3 and in particular sCD25 compared to cases with fewer or no symptoms; iv) sCD25 correlated with SARS-CoV-2 antibody titers and microneutralization titers only in cases with PCC symptoms while sTIM-3 correlated with these parameters irrespectively of symptoms. In line with this, sCD25 correlated with the breadth of CD4^+^ T cell responses in participants with symptoms, whereas sTIM-3 correlated with CD4^+^ T cell breadth and depth in patients without PCC symptoms. (v) Finally, despite markedly elevated levels of T cell activation/exhaustion markers in hospitalized patients, no relation to PCC symptoms were observed. Our study indicates a role for T cell activation and exhaustion in PCC following mild COVID-19 infection, with somewhat different patterns for sCD25, sTIM-3 and sLAG3.

Several studies report sustained T cell activation in PCC following severe COVID-19 (6–8) and indeed, hospitalized cases in our study had higher levels of all examined T cell markers (i.e., sCD25, sTIM-3 and sLAG-3) up to 12 months post-infection as compared with home-isolated patients. However, despite having more PCC-related symptoms, the association between these markers and persistent symptoms was lacking in hospitalized compared to home-isolated cases, although caution is needed when interpreting these patterns due to low numbers of severe cases. This could potentially reflect that disease severity *per se* predispose to PCC as suggested by others (19), involving a multitude of mediators not evaluated in our study. Still, in an outpatient cohort 75 days after diagnosis, Townsend et al. reported markedly higher sCD25 in hospitalized patients compared to a mild disease course but found that persistent poor health after COVID-19 was not associated with initial disease severity (20). Thus, the complex role of initial disease severity in relation to PCC is still not clear, but we hypothesize that PCC symptoms in hospitalized COVID-19 patients are more related to general disease severity than one particular mediator.

We recently reported that an overall 46% of the current population had persisting symptoms, in particular fatigue, 12 months after COVID-19 and a markedly higher risk of symptoms compared to SARS-CoV-2 negative controls (11). Herein we show sustained higher levels of sCD25 (60-74% higher) in home-isolated mild COVID-19 disease, reporting dyspnea and fatigue symptoms up to 18-months post-infection. A similar pattern was seen for sTIM-3, but with a more modest association with dyspnea and fatigue, and sLAG-3 correlated with neurocognitive symptoms towards the end of follow-up (i.e., 12 and 18 months). There are some reports of T cell subsets and T cell receptor profiles, including regulation of CD25, as well as T-cell derived cytokines in relation to PCC symptoms (6–8). Phetsouphanh et al. demonstrated elevated levels of sTIM-3 in 31 PCC patients, mostly home-isolated, four month after the infection (21). We found no studies evaluating sLAG-3 in the context of PCC. However, a concerning finding in our cohort was the high proportion of cases with cognitive symptoms at 18 months (11). Interestingly, LAG-3 is potentially expressed in the central nervous system including microglia cells (22), which potentially could be of relevance in relation to the association of sLAG-3 with memory impairment. Furthermore, sLAG-3 is elevated in patients with Parkinson’s disease and correlates with neurocognitive symptoms (23, 24). A proposed mechanism is that LAG-3 may bind and contribute to aggregation and spread of misfolded α-synuclein (25), and indices of accelerated aggregation of α-synuclein have been noted in young patients with PCC (26). Moreover, elevated sCD25 and sTIM-3 are not restricted to PCC, but reported as markers of T cell activation in various inflammatory and autoimmune disorders (27, 28) as well as some brain disorders (29). Importantly, we adjusted for several relevant co-morbidities in the present study.

The reasons for the somewhat different profiles of the three soluble markers of T cell activation/exhaustion in relation to PCC symptoms are at present not clear. While the levels of the soluble markers are thought to reflect the levels of their membrane-bound counterpart, the function of these soluble receptors is still somewhat unclear (30, 31). Still, our data underscore that the long-term impact of immune responses and T cell activation specifically, as reflected by high sCD25 and to some degree sTIM-3 and sLAG-3, on physical symptoms could be partly distinct following mild SARS-CoV-2 infection. This could impact prevention and treatment strategies targeting T cell activation in PCC, and these readily measurable soluble markers could potentially be used for risk prediction of PCC development.

We have previously shown an association between SARS-COV-2 spike-specific IgG antibody titers as well as spike-specific clonal CD4^+^ T cell responses and persistent dyspnea and symptoms at 6 and 12 months in this cohort (11). Our finding that sCD25 correlated with SARS-CoV-2 spike-specific IgG responses in patients with PCC symptoms may suggest that sCD25 to some degree could mirror the magnitude of SARS-CoV-2 specific responses following mild COVID-19. This pattern could potentially reflect a bidirectional interaction between B cells and T cells in relation to PCC, and indeed, T cell derived cytokines with activating effects on B cells have been related to PCC (31). In contrast, the correlation of sTIM-3 with SARS-CoV-2 spike-specific IgG antibody titers and CD4^+^ T cell clonal breadth and depth were largely driven by non-symptomatic participants. Thus, whereas sCD25 may specifically reflect T cell activation, sTIM-3 could partly be released from other cells (monocytes and NK-T cells) (32). Nevertheless, in the context of T cell activation, enhanced TIM-3 seems to be specific for CD4^+^ T cells and the inflammatory Th1 and Th17 subtypes in particular (33). Furthermore, it is unlikely that all T cell responses in COVID-19 are SARS-CoV-2 derived and we speculate that those reflected by sTIM-3 may mirror other T cell driven responses that could be related to PCC, but these were not included in the SARS-CoV-2 specific T cell analyses in the present study. The more prominent association between sTIM-3 levels and symptom burden at 6 and 12 months, as also seen for sCD25, may still reflect a more disproportionate T cell activation leading to a state of exhaustion (34).

The present study has some limitations. Correlations do not necessarily imply any causal relationship, and more mechanistic studies are needed to further explore the role of T cells in PCC. Although validated questionnaires were used, self-reporting scores have some inherent weaknesses. Moreover, the present cohort was infected with the Wuhan strain, and if our findings could also be applied to other SARS-CoV-2 variants need to be investigated. Moreover, all the patients in the present study were unvaccinated, and the role of SARS-CoV-2 vaccination in relation to PCC development could therefore not be examined. Also, the number of hospitalized patients and in particular the number of patients with severe disease was rather low. Strengths of study include longitudinal data on symptom scores, serum levels of T cell activation markers, antibody titers and T cell clonal responses. Moreover, the study included a near-complete geographical cohort from the first pandemic wave and the personalized follow-up to detect long COVID symptoms, which may be missed in healthcare-based registry studies.

In conclusion, we found that circulating markers of T cell activation/exhaustion were significantly associated with persistent PCC following a mild (home-isolated), but not a severe (hospitalized) SARS-CoV-2 infection where disease severity may be of more importance. In particular, a sustained increase in sCD25 was observed in relation to dyspnea and fatigue up to 18 months following infection while sTIM-3 was related to high symptom burden and sLAG-3 to long-term memory problems. Further studies are needed to pinpoint the precise mechanisms on how sustained T cell activation may promote PCC following a mild COVID-19 infection and determine if sCD25, sTIM-3 or sLAG-3 could be useful markers for risk prediction, potentially identifying individuals that could benefit from prevention and treatment strategies targeting T cell activation.

## Data Availability

For reasons related to Norwegian legislation and the participant consent forms, the data used in this article are not available in public repositories. The data are however available upon reasonable request to the corresponding author, following the establishment of a material and data transfer agreement between the institutions and the approval of an amendment application to the Regional Committee for Medical and Health Research Ethics to ensure that the aim of the planned research is covered by the participant consent forms.
