# Supplementary material for: Markers of T cell activation and exhaustion in plasma are associated with persistent symptoms up to 18 months following mild SARS-CoV-2 infection
Source: Front Immunol. 2025 May 30;16:1578208. doi: 10.3389/fimmu.2025.1578208 (PMC12162895; doi:10.3389/fimmu.2025.1578208)
Supplement: Supplementary file 1 [file DataSheet1.docx]

**Supplemental File**

***Markers of T cell activation and exhaustion in plasma are associated with persistent symptoms up to 18 months Following Mild SARS-CoV-2 infection***

***Ueland et al.***

**Supplemental Table S1. T cell activation/exhaustion markers in non-hospitalized COVID-19 cases during long-term follow-up in relation to persisting symptoms.**

|  |  | sCD25 (ng/mL) | | sTIM-3 (ng/mL) | | sLAG-3 (ng/mL) | |
| --- | --- | --- | --- | --- | --- | --- | --- |
|  | M | No symptom | Symptom | No symptom | Symptom | No symptom | Symptom |
| Dyspnea | 6 | 0.25 (0.22-0.29) | 0.44 (0.31-0.62)** | 2.70 (2.54-2.87) | 3.10 (2.67-3.61) | 0.67 (0.60-0.76) | 0.70 (0.52-0.93) |
|  | 12 | 0.24 (0.21-0.28) | 0.42 (0.29-0.59)** | 2.72 (2.58-2.86) | 2.98 (2.62-3.37) | 0.71 (0.64-0.78) | 0.93 (0.74-1.18) |
|  | 18 | 0.25 (0.21-0.30) | 0.40 (0.28-0.58)* | 2.80 (2.65-2.96) | 3.21 (2.84-3.61)* | 0.64 (0.55-0.73) | 0.82 (0.60-1.13) |
| Fatigue | 6 | 0.23 (0.20-0.28) | 0.36 (0.28-0.45)** | 2.65 (2.47-2.85) | 2.95 (2.68-3.25)** | 0.65 (0.57-0.75) | 0.72 (0.60-0.87) |
|  | 12 | 0.23 (0.19-0.27) | 0.35 (0.27-0.44)** | 2.61 (2.45-2.77) | 3.03 (2.79-3.28) | 0.72 (0.64-0.81) | 0.77 (0.66-0.90) |
|  | 18 | 0.25 (0.21-0.30) | 0.33 (0.25-0.44) | 2.84 (2.67-3.02) | 2.92 (2.67-3.21) | 0.63 (0.54-0.73) | 0.76 (0.60-0.96) |
| Impaired  concentration | 6 | 0.26 (0.22-0.30) | 0.34 (0.25-0.46) | 2.74 (2.57-2.92) | 2.79 (2.45-3.18) | 0.65 (0.58-0.74) | 0.78 (0.61-1.00) |
|  | 12 | 0.25 (0.21-0.29) | 0.32 (0.24-0.42) | 2.72 (2.57-2.88) | 2.86 (2.59-3.14) | 0.71 (0.64-0.79) | 0.83 (0.69-0.99) |
|  | 18 | 0.27 (0.22-0.32) | 0.30 (0.21-0.41) | 2.88 (2.72-3.05) | 2.81 (2.53-3.13) | 0.64 (0.55-0.74) | 0.75 (0.57-0.99) |
| Memory  problems | 6 | 0.26 (0.22-0.30) | 0.34 (0.25-0.46) | 2.74 (2.57-2.92) | 2.81 (2.46-3.20) | 0.65 (0.58-0.73) | 0.79 (0.62-1.01) |
|  | 12 | 0.25 (0.21-0.29) | 0.31 (0.24-0.41) | 2.71 (2.56-2.86) | 2.88 (2.62-3.17) | 0.69 (0.62-0.77) | 0.87 (0.73-1.04)* |
|  | 18 | 0.25 (0.21-0.30) | 0.33 (0.25-0.43) | 2.82 (2.65-3.00) | 2.98 (2.72-3.26) | 0.59 (0.51-0.69) | 0.85 (0.68-1.07)* |

s, soluble; TIM-3, T-cell immunoglobulin and mucin domain 3; LAG-3, Lymphocyte-activation gene 3 *p<0.05, **p<0.01 vs. no symptoms. Log10 transformed data were analyzed by multivariate GLM adjusted for age, sex and comorbidities. Data are shown as back-transformed estimated marginal means and 95% CI.

**Supplemental Table S2. T cell activation/exhaustion markers in non-hospitalized COVID-19 cases in relation to composite scores of persisting symptoms**

|  | Symptoms | 6 months | 12 months | 18 months |
| --- | --- | --- | --- | --- |
| sCD25  (ng/mL) | 0 | 0.24 (0.20-0.28) | 0.23 (0.19-0.28) | 0.24 (0.19-0.29) |
|  | 1 | 0.28 (0.22-0.35) | 0.27 (0.21-0.35) | 0.31 (0.23-0.42) |
|  | 2 | 0.44 (0.31-0.63)***# | 0.40 (0.29-0.54)** | 0.35 (0.25-0.50) |
| sTIM-3  (ng/mL) | 0 | 2.70 (2.51-2.92) | 2.66 (2.49-2.84) | 2.79 (2.60-2.99) |
|  | 1 | 2.64 (2.38-2.94) | 2.70 (2.46-2.96) | 2.94 (2.66-3.24) |
|  | 2 | 3.22 (2.77-3.74)*# | 3.11 (2.79-3.48)*# | 3.02 (2.68-3.40) |
| sLAG-3  (ng/mL) | 0 | 0.62 (0.54-0.72) | 0.69 (0.61-0.79) | 0.59 (0.49-0.70) |
|  | 1 | 0.75 (0.62-0.92) | 0.72 (0.61-0.86) | 0.73 (0.57-0.94) |
|  | 2 | 0.76 (0.57-1.00) | 0.91 (0.74-1.12)* | 0.85 (0.62-1.15)* |

s, soluble; TIM-3, T-cell immunoglobulin and mucin domain 3; LAG-3, Lymphocyte-activation gene 3 *p<0.05, **p<0.01, ***p<0.001 vs. no symptoms; #p<0.05 vs. 1 symptom. Log10 transformed data were analyzed by multivariate GLM adjusted for age, sex and comorbidities. Data are shown as back-transformed estimated marginal means and 95% CI.

**Supplemental Table S3. Antibody titers and T cell clonal responses in non-hospitalized COVID-19 cases according to having symptoms (S+) or not (S-).**

|  |  | 4-6 weeks | | | 6 months | | | 12 months | | |
| --- | --- | --- | --- | --- | --- | --- | --- | --- | --- | --- |
|  |  | median | 25% | 75% | median | 25% | 75% | median | 25% | 75% |
| IgG spike | All | 4104 | 1878 | 7762 | 2618 | 1115 | 5136 | 1953 | 787 | 3862 |
|  | S- | 4032 | 1812 | 7415 | 2258 | 1049 | 5078 | 1595 | 783 | 3554 |
|  | S+ | 4574 | 2179 | 8316 | 2950 | 1353 | 5241 | 2120 | 793 | 4674 |
| Microneut. titer | All | 90 | 46 | 175 | 79 | 36 | 144 | 73 | 34 | 136 |
|  | S- | 101 | 51 | 161 | 77 | 35 | 128 | 77 | 37 | 144 |
|  | S+ | 79 | 41 | 196 | 80 | 40 | 158 | 67 | 34 | 119 |
| Breadth CD4 | All | 2.6E-04 | 1.8E-04 | 4.2E-04 | 1.4E-04 | 9.8E-05 | 2.3E-04 | 2.6E-04 | 1.7E-04 | 4.2E-04 |
|  | S- | 2.3E-04 | 1.9E-04 | 3.8E-04 | 1.4E-04 | 9.9E-05 | 2.3E-04 | 2.5E-04 | 1.8E-04 | 3.8E-04 |
|  | S+ | 2.8E-04 | 1.7E-04 | 4.7E-04 | 1.4E-04 | 9.4E-05 | 2.3E-04 | 2.7E-04 | 1.7E-04 | 4.7E-04 |
| Depth CD4 | All | 66 | 36 | 112 | 24 | 6 | 51 | 66 | 36 | 112 |
|  | S- | 65 | 41 | 112 | 19 | 4 | 40 | 68 | 40 | 103 |
|  | S+ | 70 | 35 | 112 | 32 | 12 | 60 | 62 | 29 | 112 |
| Breadth CD8 | All | 2.7E-05 | 1.5E-05 | 6.0E-05 | 1.3E-05 | 5.0E-06 | 3.5E-05 | 2.7E-05 | 1.5E-05 | 6.0E-05 |
|  | S- | 2.3E-05 | 1.4E-05 | 5.3E-05 | 1.2E-05 | 4.4E-06 | 3.5E-05 | 2.6E-05 | 1.4E-05 | 6.5E-05 |
|  | S+ | 3.9E-05 | 2.2E-05 | 7.2E-05 | 1.5E-05 | 7.1E-06 | 3.3E-05 | 2.7E-05 | 1.8E-05 | 5.7E-05 |
| Depth CD8 | All | -8.8 | -13.8 | 1 | -13.8 | -16.5 | -7.4 | -8.8 | -13.8 | 0.8 |
|  | S- | -10.5 | -14.5 | -0.7 | -14.1 | -16.6 | -9.4 | -9.8 | -14.1 | 5.4 |
|  | S+ | -6.8 | -12.2 | 2.1 | -13.1 | -16.3 | -6.4 | -8.1 | -13.7 | -0.9 |
| Breadth spike CD4 | All | 4.1E-05 | 2.3E-05 | 7.5E-05 | 2.1E-05 | 1.0E-05 | 3.7E-05 | 4.1E-05 | 2.3E-05 | 7.5E-05 |
|  | S- | 3.8E-05 | 2.3E-05 | 6.6E-05 | 2.1E-05 | 1.0E-05 | 3.8E-05 | 3.9E-05 | 2.6E-05 | 6.7E-05 |
|  | S+ | 4.6E-05 | 2.5E-05 | 7.8E-05 | 2.2E-05 | 1.1E-05 | 3.7E-05 | 4.6E-05 | 2.2E-05 | 7.6E-05 |
| Depth spike CD4 | All | -5.1 | -11 | 3.5 | -12.1 | -14.7 | -6.1 | -5.2 | -11.3 | 3.5 |
|  | S- | -6 | -12.2 | 2.7 | -13.1 | -14.8 | -8.3 | -5.4 | -11.9 | 3 |
|  | S+ | -3.4 | -10.7 | 5.7 | -10.5 | -14.4 | -5.4 | -5.2 | -10.8 | 3.8 |
| Breadth spike CD8 | All | 0.0E+00 | 0.0E+00 | 4.1E-06 | 0.0E+00 | 0.0E+00 | 2.5E-06 | 0.0E+00 | 0.0E+00 | 4.1E-06 |
|  | S- | 0.0E+00 | 0.0E+00 | 3.5E-06 | 0.0E+00 | 0.0E+00 | 1.6E-06 | 0.0E+00 | 0.0E+00 | 4.4E-06 |
|  | S+ | 0.0E+00 | 0.0E+00 | 4.9E-06 | 0.0E+00 | 0.0E+00 | 2.8E-06 | 0.0E+00 | 0.0E+00 | 4.1E-06 |
| Depth spike CD8 | All | -18.1 | -18.8 | -16.7 | -18.3 | -19 | -17.4 | -18.1 | -18.8 | -16.7 |
|  | S- | -18.2 | -19 | -17.1 | -18.1 | -19.1 | -17.1 | -18.2 | -19 | -16.8 |
|  | S+ | -18 | -18.6 | -16.3 | -18.5 | -19 | -17.7 | -18 | -18.7 | -16.5 |
| Breadth nonspike CD4 | All | 5.1E-05 | 3.0E-05 | 8.8E-05 | 2.8E-05 | 1.7E-05 | 4.6E-05 | 5.1E-05 | 3.0E-05 | 8.8E-05 |
|  | S- | 5.1E-05 | 3.1E-05 | 9.0E-05 | 2.8E-05 | 1.8E-05 | 4.6E-05 | 5.5E-05 | 3.3E-05 | 9.1E-05 |
|  | S+ | 5.1E-05 | 2.9E-05 | 8.0E-05 | 2.7E-05 | 1.5E-05 | 4.5E-05 | 4.8E-05 | 2.6E-05 | 7.6E-05 |
| Depth nonspike CD4 | All | -1.7 | -8.8 | 7.4 | -10.9 | -13.4 | -4.6 | -1.7 | -8.8 | 7.4 |
|  | S- | -1.7 | -8.2 | 6.8 | -11.2 | -13.5 | -5.2 | 0.7 | -7 | 7.9 |
|  | S+ | -1.6 | -9.1 | 7.9 | -9.4 | -13.3 | -2.6 | -3.1 | -10.1 | 6.8 |
| Breadth nonspike CD8 | All | 7.5E-06 | 0.0E+00 | 3.9E-05 | 4.6E-06 | 0.0E+00 | 1.8E-05 | 7.3E-06 | 0.0E+00 | 3.9E-05 |
|  | S- | 5.5E-06 | 0.0E+00 | 3.6E-05 | 4.6E-06 | 0.0E+00 | 1.7E-05 | 6.1E-06 | 0.0E+00 | 4.3E-05 |
|  | S+ | 1.1E-05 | 2.7E-06 | 4.5E-05 | 4.9E-06 | 0.0E+00 | 1.9E-05 | 9.2E-06 | 0.0E+00 | 3.5E-05 |
| Depth nonspike CD8 | All | -15.9 | -18 | -7 | -16.5 | -18.1 | -13.1 | -15.8 | -18 | -7.4 |
|  | S- | -16.2 | -18.2 | -9.3 | -16.6 | -18.1 | -13.6 | -16.1 | -18.1 | -2.7 |
|  | S+ | -14.2 | -17.4 | -5.4 | -16.4 | -18.2 | -12.6 | -15.4 | -17.5 | -9.3 |

Data are given as median and 25^th^ and 75^th^ percentiles.

**
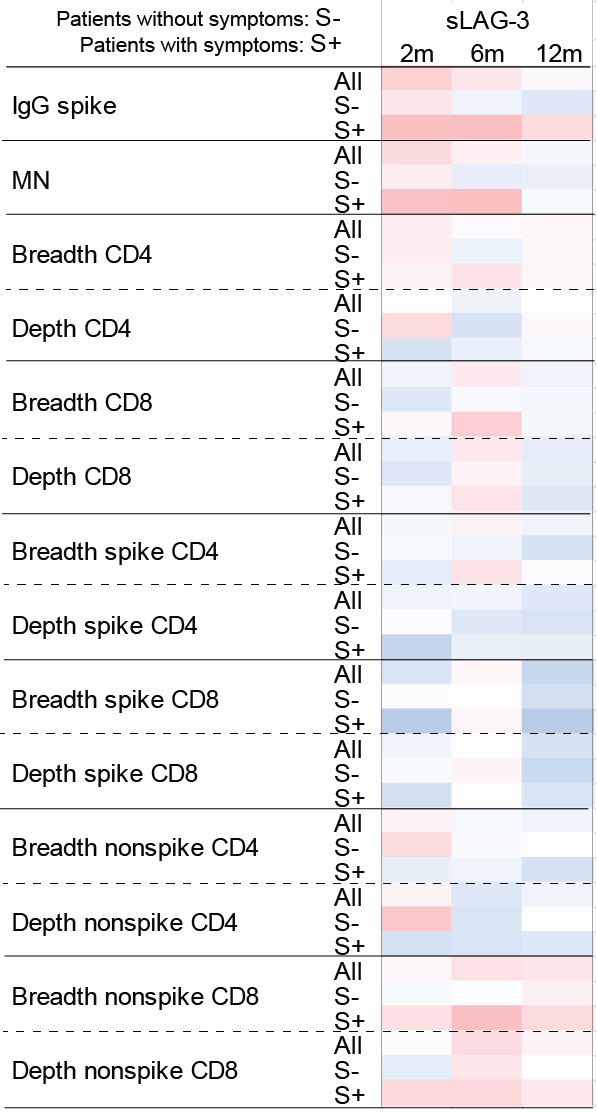
**

**Supplemental Figure 1. Associations between sLAG-3, antibody titers and T cell clonal response. A)** Heatmap showing correlations between sLAG-3 and IgG spike and microneutralization titers and TCRβ SARS-CoV-2 spike specific and non-spike specific CD4^+^ and CD8^+^ T cell clonal depth and breadth at different time-points during follow-up in all home-isolated COVID-19 cases (All: in all patients) and in relation to having (S+) or not having any symptom (S-) at the same time-points. For correlations at 2 months, 6-month symptoms were used. Spearman correlation coefficients are included for significant correlations.
